# Point-of-care testing of coagulation in patients treated with edoxaban

**DOI:** 10.1007/s11239-020-02143-2

**Published:** 2020-05-20

**Authors:** Florian Härtig, Ingvild Birschmann, Andreas Peter, Sebastian Hörber, Matthias Ebner, Matthias Sonnleitner, Charlotte Spencer, Paula Bombach, Maria-Ioanna Stefanou, Joachim Kuhn, Annerose Mengel, Ulf Ziemann, Sven Poli

**Affiliations:** 1 Department of Neurology & Stroke, and Hertie Institute for Clinical Brain Research, Eberhard-Karls University, Tübingen, Tübingen, Germany; 2 Institute for Laboratory and Transfusion Medicine, Heart and Diabetes Center, Ruhr University, Bad Oeynhausen, Germany; 3 Department for Diagnostic Laboratory Medicine, Institute for Clinical Chemistry and Pathobiochemistry, University Hospital Tuebingen, Tübingen, Germany; 4 Department of Nephrology and Medical Intensive Care, Charité – University Medicine Berlin, Berlin, Germany

**Keywords:** Point-of-care, CoaguChek, DOAC, Stroke, Thrombolysis, Anticoagulation reversal

## Abstract

Edoxaban, alongside other direct oral anticoagulants (DOAC), is increasingly used for prevention of thromboembolism, including stroke. Despite DOAC therapy, however, annual stroke rate in patients with atrial fibrillation remains 1–2%. Rapid exclusion of relevant anticoagulation is necessary to guide thrombolysis or reversal therapy but, so far, no data exists on the effect of edoxaban on available point-of-care test systems (POCT). To complete our previous investigation on global coagulation-POCT for the detection of DOAC, we evaluated whether CoaguChek®-INR (CC-INR) is capable of safely ruling out edoxaban concentrations above the current treatment thresholds of 30/50 ng/mL in a blood sample. We studied patients receiving a first dose of edoxaban; excluding subjects receiving other anticoagulants. Six blood samples were collected from each patient: before drug intake, 0.5, 1, 2 and 8 h after intake, and at trough (24 h). CC-INR and mass spectrometry for edoxaban concentrations were performed for each time-point. One hundred and twenty blood samples from 20 patients contained 0–302 ng/mL of edoxaban. CC-INR ranged from 0.9 to 2.3. Pearson’s correlation coefficient showed strong correlation between CC-INR and edoxaban concentrations (r = 0.73, *p* < 0.001). Edoxaban concentrations > 30 and > 50 ng/mL were ruled out by CC-INR ≤ 1.0 and ≤ 1.1, respectively, with high specificity (> 95%), and a sensitivity of 44% (95%-confidence interval: 30–59%) and 86% (74–93%), respectively. Our study represents the first evaluation of coagulation-POCT in edoxaban-treated patients. CC-POCT is suitable to safely exclude clinically relevant edoxaban concentrations prior to thrombolysis, or guide reversal therapy in stroke patients.

## Highlights

- First study of point-of-care coagulation testing in edoxaban-treated patients.
- Using a sufficiently low INR cut-off, the CoaguChek® is able to safely exclude edoxaban and rivaroxaban plasma concentrations above the guideline-endorsed thresholds of 30 and 50 ng/mL.
- Thus, identification of thrombolysis eligible stroke patients, or—in case of hemorrrhage—patients without need for reversal therapy becomes feasible within a minute.

## Introduction

Alongside other direct oral anticoagulants (DOAC), edoxaban is increasingly replacing vitamin K antagonists (VKA) for treatment and prevention of thrombosis and thromboembolism including ischemic stroke [1]. Similar to the other DOAC, edoxaban has gained approval by providing comparable efficacy and improved safety. Annual risk for ischemic stroke in edoxaban-treated patients with atrial fibrillation, however, remains above 1%, and ~ 0.4% for intracranial hemorrhage [2]. In these situations, DOAC-mediated anticoagulation needs to be excluded prior to initiation of thrombolysis or in order to avoid unnecessary administration of expensive and potentially pro-thrombotic reversal therapy. (Calibrated) anti-Xa activity assays (AXA) are recommended by guidelines as state-of-the-art for coagulation assessment during edoxaban therapy [3, 4]. Unfortunately, these assays are not available on any commercial point-of-care test device (POCT), and laboratory-based coagulation testing clearly limits emergency decision making due to their long turnaround-times [5].

In analogy to our previous research conducted with apixaban, dabigatran, and rivaroxaban [6], we aimed to determine whether the commercially available prothrombin time (PT)/international normalized ratio (INR)-based CoaguChek® (CC)-POCT (Roche Diagnostics, Rotkreuz, Switzerland) allows for ruling out low but clinically relevant edoxaban plasma concentrations in a blood sample.

## Methods

### Study design

Single-center, prospective diagnostic study with partially blinded outcome assessment. Independent review board approval was obtained prior to all study-related activity from the ethics committee of Tübingen University Hospital (Protocol No. 270/2015BO1). The Clinical Trial Registration Information unique identifier for the study is NCT02825394. Written informed consent was obtained from all patients before enrollment.

### Setting and eligibility criteria

The study was conducted at the Department of Neurology of Tübingen University Hospital, a tertiary care facility in Germany. We enrolled ischemic stroke patients receiving a first dose of edoxaban for secondary prevention of thromboembolism. Subjects who had received VKA or DOAC within 14 days, low-molecular-weight heparin within 24 h, or unfractionated heparin within 12 h before first DOAC intake were excluded to rule out interference with measurements. Patients with either abnormal coagulation values at baseline (INR > 1.2 or activated partial thromboplastin time (aPTT) > 40 s), or history of coagulopathy were also excluded. Use of anti-platelet drugs was permitted.

### Sample collection and coagulation testing

Six blood samples were collected from each subject via a venous catheter or by direct venipuncture before first intake of edoxaban, and 0.5, 1, 2, and 8 h after intake, and at trough (24 h). This was done in order to cover a wide range of edoxaban concentrations.

Whole blood was drawn directly into a non-heparinized syringe (Injekt, B.Braun, Melsungen, Germany) and used to conduct CC-INR within 15 s of sampling (compare [6] for device specifications).

Additional blood was drawn into a standard blood sampling tube for coagulation assays (S-Monovette Citrate 3.2%, Sarstedt, Nümbrecht, Germany). One sample of citrated whole-blood was sent to the central laboratory of Tübingen University Hospital for laboratory-based INR (Lab-INR), aPTT (Lab-aPTT) and calibrated AXA, using the Dade Innovin, Actin FS, and Innovance Heparin assays, respectively, on a Sysmex CS-5100 (all Siemens Healthineers, Erlangen, Germany). Further samples of citrated whole blood were centrifuged at 2500×*g* for 15 min to yield citrated plasma (cp.), which was stored at − 80 °C. One cp. sample per time-point was later shipped to the Institute for Laboratory and Transfusion Medicine at the Heart and Diabetes Center of Ruhr University (Bad Oeynhausen, Germany) for ultra-performance liquid chromatography–tandem mass spectrometry (UPLC-MS/MS) as a gold-standard method to determine exact edoxaban plasma concentrations [7]. Additionally, at baseline, a full blood count, coagulation tests, inflammatory markers, protein/albumin as well as liver and kidney function tests were performed.

All POCT and laboratory-based tests were performed according to manufacturers’ instructions by thoroughly trained investigators and technicians.

### Blinding

All POCT operators were blinded to the results of all other coagulation assays as well as those of UPLC-MS/MS. External technicians conducting UPLC-MS/MS were blinded to the results of all coagulation assays including CC-INR as well as patient number and sampling time-point. Fully automated laboratory-based measurements were conducted at our central laboratory where technicians were blinded to the results of CC-INR and UPLC-MS/MS.

### Definition of relevant DOAC concentrations

Since the publication of our previous evaluation of the CC-POCT for measurement of DOAC in 2015 [6], guideline recommendations regarding the threshold allowing thrombolysis have changed. Currently, plasma concentrations ≤ 30 or ≤ 50 ng/mL are recommended for all DOAC including edoxaban  [4, 8]. The same thresholds might allow urgent surgical procedures [9]. Equally, reversal therapy in intracranial hemorrhage [4] or serious bleeding [9] respectively, should be triggered above these thresholds.

### Statistical analyses

Pearson’s correlation coefficient was used to estimate the strength of correlation between CC-INR and UPLC-MS/MS. Different CC-INR cut-offs were evaluated regarding their capability to categorize samples according to the two edoxaban concentration thresholds (≤ 30 and ≤ 50 ng/mL), up to which edoxaban levels were defined as “not clinically relevant”. Specificity was defined as the percentage of samples containing clinically relevant edoxaban concentrations that were correctly identified by elevated CC-INR values. Sensitivity was defined as the percentage of samples containing no clinically relevant edoxaban concentrations that were correctly identified as such by low CC-INR and, thus, as theoretically belonging to a patient eligible for immediate thrombolysis or surgery. Positive and negative predictive values as well as likelihood ratios (sensitivity/1 − specificity) were calculated. In analogy to other authors [10], we provide a misprediction percentage (MP = 1 − specificity), representing the frequency of unremarkable CC-INR despite relevant edoxaban concentrations. An ideal CC-INR cut-off was pre-defined by the highest possible value that yielded a specificity of > 95% (MP < 5%) in order to avoid false negatives, which might constitute a significant safety issue.

Receiver operating characteristics (ROC) curve were drawn and the area under the ROC curve (AUROC) calculated. 95%-confidence intervals (CI) for all proportions were calculated according to the efficient-score method as described by Newcombe [11] using the free online VassarStats Clinical Calculator 1 [12]. SPSS version 24 (IBM, Armonk, NY, USA) was used for all other statistical analyses. The study was performed and is reported according to STARD guidelines [13].

## Results

### Patient population

Between October 2016 and May 2017, twenty patients receiving a first dose of edoxaban for secondary stroke prevention were included (see Table 1 for baseline characteristics) leading to 120 measurements of CC-INR and edoxaban plasma concentrations (UPLC-MS/MS).

**Table 1:** Patient baseline characteristics (N = 20)

Age	66 ± 10.5 years
Female sex	8 (40%)
Edoxaban dose	60 mg daily: 15 (75%) 30 mg daily: 5 (25%)
Body weight	80.5 ± 20.11 kg
Body Mass Index (BMI)	27.0 ± 5.87 kg/m^2^
Estimated glomerular filtration rate (CDK-EPI)	74 ± 18.6 mL/min/1.73 m^2^
Estimated glomerular filtration rate (MDRD)	74 ± 21.5 mL/min/1.73 m^2^
Estimated glomerular filtration rate (Cockcroft-Gault)	83 ± 26.0 mL/min/1.73 m^2^
Risk factors	
Arterial hypertension	16 (80%)
Hyperlipidemia	7 (35%)
Diabetes mellitus	1 (5%)
History of stroke	20 (100%)
Congestive heart failure	2 (10%)
Coronary heart disease	7 (35%)
History of myocardial infarction	5 (25%)
Smoking	4 (20%)
Indication for anticoagulation therapy	
Atrial fibrillation (AF)	12 (60%)
Stroke associated with patent foramen ovale	6 (30%)
Embolic stroke of undetermined source (off label)	2 (10%)
Concomitant antiplatelet therapy	4 (20%)

Continuous variables are displayed as mean ± standard deviation

Nominal variables are displayed as absolute quantity (percentage)

### Test results and correlation between CC-INR and edoxaban concentrations

Edoxaban plasma concentrations ranged from 0 to 302 ng/mL, whilst 50 of the 120 samples (42%) contained ≤ 30 ng/mL and 70 samples (58%) contained > 30 ng/mL of edoxaban. 57 samples (47.5%) contained ≤ 50 ng/mL and 63 samples (52.5%) contained > 50 ng/mL. CC-INR ranged from 0.9 to 2.3 and correlated well with actual edoxaban concentrations (r = 0.73, *p* < 0.001).

### Diagnostic accuracy of POCT to detect relevant edoxaban concentrations

**Fig. 1:**
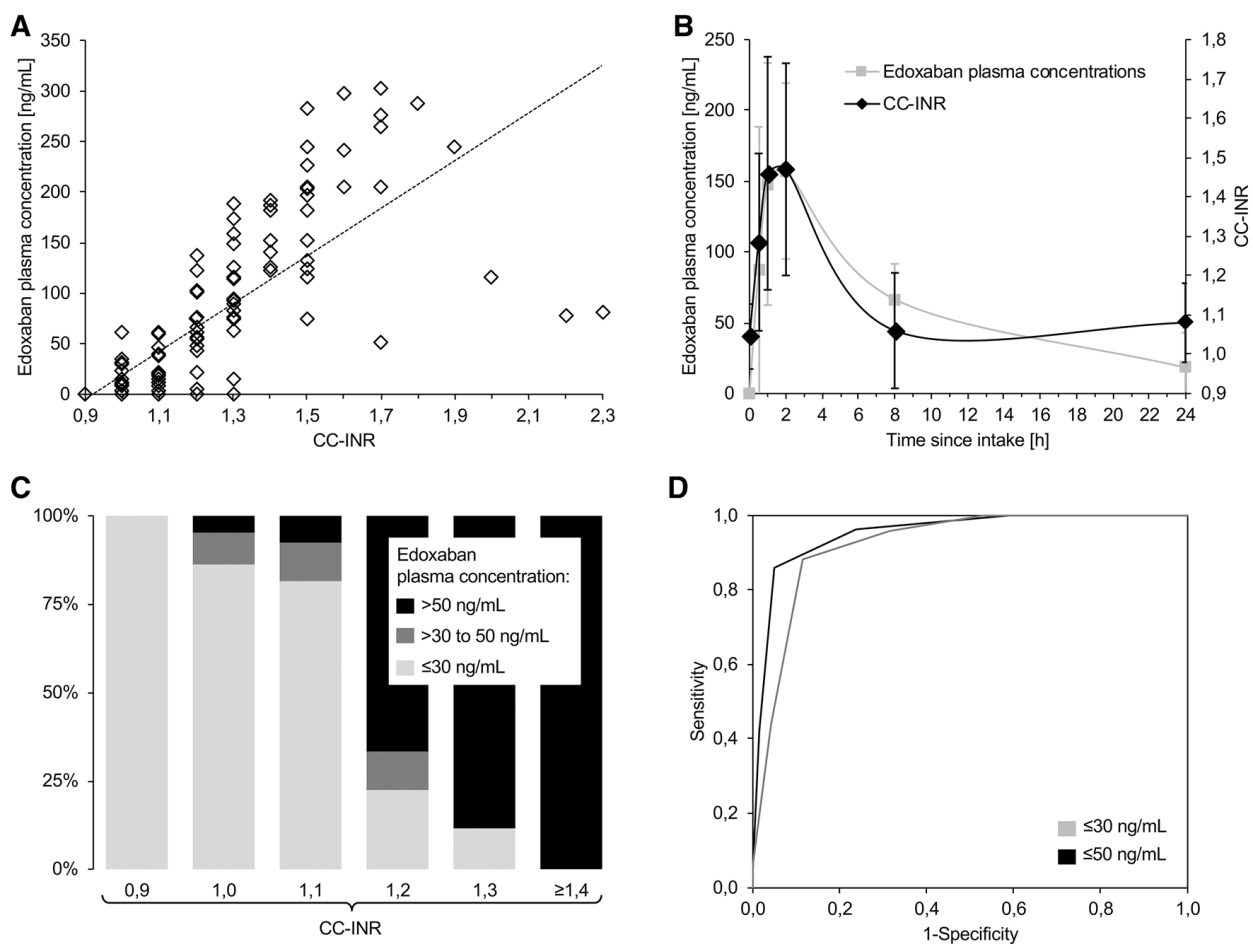
**a** Correlation of CoaguChek®-international normalized ratio (CC-INR) values and edoxaban plasma concentrations (dashed line: regression line), **b** changes in edoxaban plasma concentrations and CC-INR results over the course of the study period (displayed as mean ± one standard deviation), **c** percentage of edoxaban plasma concentrations below and above the treatment-relevant thresholds of 30 and 50 ng/mL found at different CC-INR levels, and **d** receiver operating characteristics curve found for CC-INR when testing for detection of samples containing edoxaban plasma concentrations ≤ 30 and ≤ 50  ng/mL

All data regarding diagnostic accuracy and ROC of CC-INR are summarized in Table 2; Fig. 1. At the 30 ng/mL-threshold, CC-INR reached an AUROC of 0.92 (95% CI 0.88–0.97). The ideal cut-off was found at a CC-INR value of ≤ 1.0. At the 50 ng/mL-threshold, CC-INR reached an AUROC of 0.96 (95% CI 0.92–0.99). The ideal cut-off was found at a CC-INR value of ≤ 1.1.

**Table 2:** Diagnostic accuracy of the CoaguChek®-international normalized ratio (CC-INR) regarding detection of edoxaban (and rivaroxaban) plasma concentrations ≤ 30 and ≤ 50 ng/mL

Threshold (ng/mL)	Cut-off	Specificity (%)	Sensitivity (%)	MP (%)	LR (%)	PPV (%)	NPV (%)
Edoxaban ≤ 30	CC-INR ≤ 1.0	95.7 (87.2–98.9)	44.0 (30.3–58.7)	4.3	10.27 (3.25–32.44)	88.0 (67.7–96.8)	70.5 (60.2–79.2)
	**CC-INR ≤ 1.1**	**88.6 (78.2–94.6)**	**88.0 (0.75–95.0)**	**11.4**	**7.70 (3.98–14.90)**	**84.6 (71.4–92.7)**	**91.2 (81.1–96.4)**
	**CC-INR ≤ 1.2**	**68.6 (56.2–78.9)**	**96.0 (85.1–99.3)**	**31.4**	**3.05 (2.15–4.34)**	**68.6 (56.2–78.9)**	**96.0 (85.1–99.3)**
	Cal. AXA ≤ 30 ng/mL	100 (93.5–100)	92.0 (79.9–97.4)	0	–	100 (90.4–100)	94.6 (86.0–98.3)
	**Lab-INR normal**	**47.8 (35.8–60.1)**	**92.0 (79.9–97.4)**	**52.2**	**1.8 (1.4–2.2)**	**56.1 (44.7–66.9)**	**89.2 (73.6–96.5)**
	**Lab-aPTT normal**	**0.0 (0.0–6.6)**	**100 (91.1–100)**	**100**	**1.0 (1.0–1.0)**	**42.0 (33.1–51.4)**	**–**
Edoxaban ≤ 50	CC-INR ≤ 1.0	98.4 (90.3–99.9)	42.1 (29.4–55.9)	1.6	26.52 (3.71–189.84)	96.0 (77.7–99.8)	65.3 (54.7–74.5)
	CC-INR ≤ 1.1	95.2 (85.8–98.8)	86.0 (73.7–93.3)	4.8	18.05 (5.95–54.74)	94.2 (83.1–98.5)	88.2 (77.6–94.4)
	**CC-INR ≤ 1.2**	**76.2 (63.5–85.6)**	**96.5 (86.8–99.4)**	**23.8**	**4.05 (2.60–6.32)**	**78.6 (66.8–87.1)**	**96.0 (85.1–99.3)**
	Cal. AXA ≤ 50 ng/mL	98.4 (90.3–99.9)	87.7 (75.7–94.5)	1.6	55.26 (7.89–387.17)	98.0 (88.2–99.9)	89.9 (79.6–95.5)
	**Lab-INR normal**	**53.2 (40.2–65.8)**	**93.0 (82.2–97.7)**	**46.8**	**2.0 (1.5–2.6)**	**64.6 (53.2–74.7)**	**89.2 (73.6–96.5)**
	**Lab-aPTT normal**	**0.0 (0.0–7.3)**	**100 (92.1–100)**	**100**	**1.0 (1.0–1.0)**	**47.9 (38.7–57.2)**	**–**
Rivaroxaban ≤ 30	CC-INR ≤ 0.9	98.7 (91.9–99.9)	11.9 (4.5–26.4)	1.3	9.05 (1.09–74.90)	83.3 (36.5–99.1)	67.0 (57.4–75.4)
	**CC-INR ≤ 1.0**	**87.9 (77.0–94.3)**	**71.4 (55.2–83.8)**	**12.1**	**5.89 (2.99–11.60)**	**78.9 (62.2–89.9)**	**82.9 (71.6–90.5)**
Rivaroxaban ≤ 50	CC-INR ≤ 0.9	100 (93.4–100)	12.2 (5.1–25.5)	0	-	100 (51.7–100)	61.6 (51.9–70.5)
	**CC-INR ≤ 1.0**	**91.3 (81.4–96.4)**	**65.3 (50.3–77.9)**	**18.7**	**7.51 (3.40–16.57)**	**84.2 (68.1–93.4)**	**78.8 (67.9–86.8)**

Specificity, sensitivity, MP, PPV, and NPV are displayed in % with 95%-confidence intervals in brackets. LR is displayed with 95%-confidence intervals in brackets. Rows are bolded if target-specificity of > 95% is not reached. Calculations of CC-INR cut-offs for rivaroxaban are based on a re-analysis of sample-level data originally published in [6]

*Lab-aPTT* laboratory-based activated partial thromboplastin time, *Cal. AXA* calibrated laboratory-based anti-Xa activity, *CC-INR* CoaguChek-based INR, *Lab-INR* laboratory-based INR, *LR* likelihood ratio, *MP* misprediction percentage, *NPV* negative predictive value, *PPV* positive predictive value

### Diagnostic accuracy of laboratory-based Anti-Xa activity

All data regarding diagnostic accuracy of calibrated AXA are summarized in Table 2.

The AUROC was 1.00 (95% CI 1.00–1.00) at the 30 ng/mL-threshold, and 0.99 (0.98–1.00) at the 50 ng/mL-threshold.

### Laboratory-based global coagulation tests: correlation with edoxaban concentrations and diagnostic accuracy

Lab-INR and Lab-aPTT also correlated well with actual edoxaban concentrations (r = 0.76 and 0.88 respectively, both p < 0.001). However, neither Lab-INR nor Lab-aPTT values within the normal range reached our specificity target of 95% for ruling out low edoxaban concentrations (see Table 2).

## Discussion

In this study, we were able to demonstrate that CC-INR correlates well with actual edoxaban plasma concentrations and thus, may be used to exclude clinically relevant edoxaban concentrations in real-life blood samples by applying CC-INR-specific cut-offs for each threshold. Note, that these cut-offs lie below the upper limit of the CC-INR’s normal range of ≤ 1.2. In this study, we tested around two edoxaban thresholds, which have been established by experts and endorsed by clinical guidelines [4, 8, 9]. Both thresholds are, however, not yet supported by prospective clinical data.

**Fig. 2:**
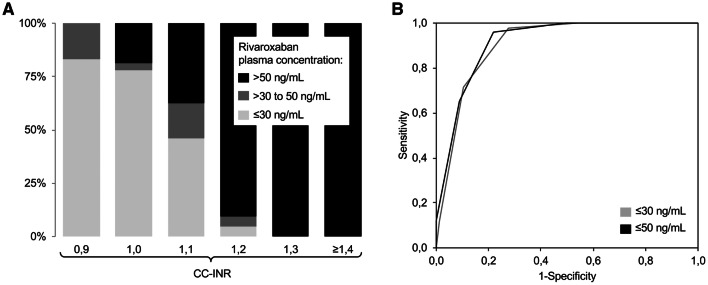
**a** Percentage of rivaroxaban plasma concentrations below and above the treatment-relevant thresholds of 30 and 50 ng/mL found at different level of CoaguChek®-international normalized ratio (CC-INR), and **b** receiver operating characteristics curve found for CC-INR when testing for detection of samples containing rivaroxaban plasma concentrations ≤ 30 and ≤ 50 ng/mL

Based on the data collected during our original study [6], we re-calculated the diagnostic accuracy of CC-INR for rivaroxaban samples at the current thresholds (see Table 2; Fig. 2). The lower ideal CC-INR cut-off of only 0.9 (for both thresholds) indicates a weaker effect of rivaroxaban on CC-INR than edoxaban (the AUROC was 0.90 (95% CI 0.85–0.96) at the 30 ng/mL-threshold and 0.92 (95% CI 0.87–0.97) at the 50 ng/mL-threshold). It is notable that rivaroxaban plasma concentrations below both thresholds are ideally detected using a cut-off of 0.9. This is due to the two suggested thresholds—30 and 50 ng/mL—being low and rather close together. Additionally, the dataset in this area was limited with only seven samples containing > 30 and ≤ 50 ng/mL of rivaroxaban.

Furthermore, we have found ‘normal’ Lab-INR and Lab-aPTT to be insufficient for the exclusion of edoxaban plasma concentrations at the proposed thresholds despite good linear correlation. Clearly, use of the normal reference range greatly underestimates edoxaban plasma concentrations as shown in Table 2. Consequently, individual (lowered) cut-offs could be defined as we have done for CC-INR. Given the known variable effect of edoxaban on different PT and aPTT assays [14], however, this is not a pragmatic approach as the individual cut-offs need to be established for each individual assay. Therefore, the use of laboratory-based global coagulation tests for the identification of patients eligible for thrombolysis is generally not recommendable as discussed in more detail in a previous publication of our group [15].

This study completes our previous evaluation of CC-INR in DOAC-treated patients: with a specificity of > 95%, CC-INR is able to safely exclude elevated plasma concentrations of not only rivaroxaban [6] but also edoxaban. Unfortunately, this does not apply to apixaban and dabigatran [6]. This is not surprising in case of the thrombin inhibitor dabigatran. For apixaban, which is—like edoxaban and rivaroxaban—an inhibitor of coagulation factor Xa, this issue has been noted in previous publications (some of which were discussed in [16]). Unfortunately, there has not yet been a conclusive explanation for this and our own data does not provide one either.

### Strengths and limitations

All coagulation testing was conducted using real-life patient samples and edoxaban concentrations around both treatment-relevant thresholds are well represented in the dataset, which supports the validity of the presented analyses. No data was lost. Edoxaban concentrations were measured using UPLC-MS/MS rather than estimated by calibrated AXA [17].

The aim of this study was to evaluate the ability of CC-INR to safely exclude treatment-relevant edoxaban concentrations in the emergency situation; analyzed samples, however, were taken from patients in a non-emergency setting. This was done for reasons of feasibility and in order to be able to analyze a wide range of edoxaban concentrations.

Sensitivity of the ideal CC-INR cut-offs achieving > 95% specificity (≤ 1.0/≤ 1.1) is limited, i.e. only 44%/86% of patients with no relevant level of anticoagulation (≤ 30/≤ 50 ng/mL) are identified as such. Higher diagnostic accuracy is achieved by using calibrated AXA. Relying solely on laboratory-based testing, however, causes significant delays in thrombolysis or urgent surgery, while clinical suspicion of DOAC-intake may lead to unnecessary and potentially harmful reversal therapy.

The cut-offs suggested in this manuscript were established retrospectively and warrant prospective clinical evaluation. Also, they are not transferable to other PT/INR-based POCT devices or laboratory-based assays, as different reagents are used [15].

It is important to note that in order to use CC-INR to exclude relevant DOAC plasma concentrations the type of DOAC and the approximate time of the last dose must be known. Otherwise, relevant DOAC concentrations (e.g. of apixaban or dabigatran [6]) may be overlooked or drug levels might still be on the rise during the first hours after intake.

## Conclusion

This study represents the first evaluation of coagulation testing in edoxaban-treated patients using a commercially available POCT and completes our evaluation of the PT/INR-based CC-POCT regarding monitoring of all four currently FDA- and EMA-approved DOAC [6].

**Fig. 3:**
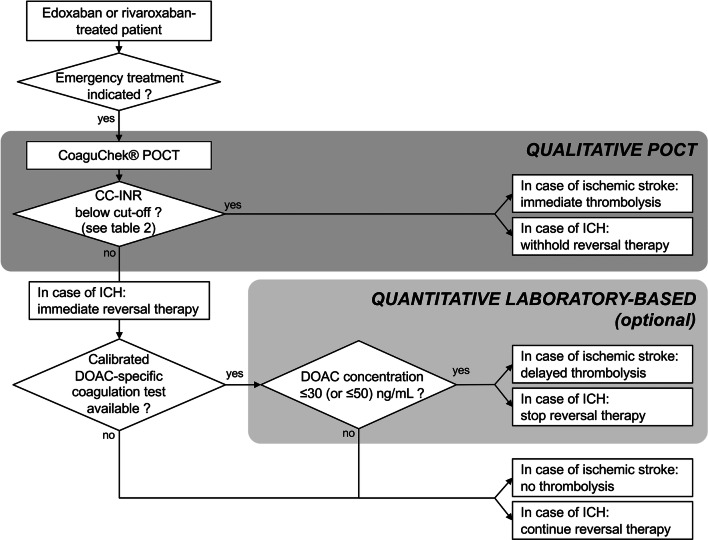
Proposed algorithm for emergency coagulation assessment using the CoaguChek® point-of-care test system (POCT) for rapid decision making in edoxaban and rivaroxaban-treated patients. *CC-INR* CoaguChek®-international normalized ratio, *DOAC* direct oral anticoagulant, *ICH* intracranial hemorrhage

Using sufficiently low CC-INR cut-offs (≤ 1.0/≤ 1.1), the CC-POCT is able to reliably rule out elevated edoxaban plasma concentrations (> 30/> 50 ng/mL) with > 95% specificity, whilst identifying patients with no relevant anticoagulation with a sensitivity of 44% and 86%, respectively. It may, thus, be used to safely identify a large fraction of edoxaban-treated patients, who might immediately receive thrombolysis in case of acute ischemic stroke or undergo urgent surgery. In patients with intracerebral hemorrhage, potentially harmful reversal therapy may be withheld (see Fig. 3 for a practical approach).

Prospective validation—ideally in the emergency situation—is warranted.
